# Mail-Based Self-Sampling to Complete Colorectal Cancer Screening: Accelerating Colorectal Cancer Screening and Follow-up Through Implementation Science

**DOI:** 10.5888/pcd20.230083

**Published:** 2023-12-07

**Authors:** Samir Gupta, Autumn Barnes, Alison T. Brenner, Janis Campbell, Melinda Davis, Kevin English, Sonja Hoover, Karen Kim, Sarah Kobrin, Peter Lance, Shiraz I. Mishra, Jill M. Oliveri, Daniel S. Reuland, Sujha Subramanian, Gloria D. Coronado

**Affiliations:** 1 University of California, San Diego, La Jolla, California; 2 Jennifer Moreno VA Healthcare System, San Diego, California; 3 RTI International, Research Triangle Park, North Carolina; 4 University of North Carolina at Chapel Hill, Lineberger Comprehensive Cancer Center, Chapel Hill, North Carolina; 5 Division of General Medicine and Clinical Epidemiology, University of North Carolina School of Medicine, Chapel Hill; 6 Department of Biostatistics and Epidemiology, Hudson College of Public Health, The University of Oklahoma Health Sciences Center, Oklahoma City; 7 Oregon Health & Science University, Portland; 8 Albuquerque Area Indian Health Board, Inc, Albuquerque, New Mexico; 9 University of Chicago Medicine, Chicago, Illinois; 10 Division of Cancer Control and Population Sciences, National Cancer Institute, Bethesda, Maryland; 11 University of Arizona Cancer Center, Tuscon; 12 University of New Mexico Comprehensive Cancer Center and Health Sciences Center, Albuquerque; 13 The Ohio State University Comprehensive Cancer Center, Columbus; 14 Kaiser Permanente Center for Health Research, Portland, Oregon

## Abstract

**Introduction:**

Leveraging cancer screening tests, such as the fecal immunochemical test (FIT), that allow for self-sampling and postal mail for screening invitations, test delivery, and return can increase participation in colorectal cancer (CRC) screening. The range of approaches that use self-sampling and mail for promoting CRC screening, including use of recommended best practices, has not been widely investigated.

**Methods:**

We characterized self-sampling and mail strategies used for implementing CRC screening across a consortium of 8 National Cancer Institute Cancer Moonshot Initiative Accelerating Colorectal Cancer Screening and Follow-up through Implementation Science (ACCSIS) research projects. These projects serve diverse rural, urban, and tribal populations in the US.

**Results:**

All 8 ACCSIS projects leveraged self-sampling and mail to promote screening. Strategies included organized mailed FIT outreach with mailed invitations, including FIT kits, reminders, and mailed return (n = 7); organized FIT-DNA outreach with mailed kit return (n = 1); organized on-demand FIT outreach with mailed offers to request a kit for mailed return (n = 1); and opportunistic FIT-DNA with in-clinic offers to be mailed a test for mailed return (n = 2). We found differences in patient identification strategies, outreach delivery approaches, and test return options. We also observed consistent use of Centers for Disease Control and Prevention Summit consensus best practice recommendations by the 7 projects that used mailed FIT outreach.

**Conclusion:**

In research projects reaching diverse populations in the US, we observed multiple strategies that leverage self-sampling and mail to promote CRC screening. Mail and self-sampling, including mailed FIT outreach, could be more broadly leveraged to optimize cancer screening.

**Summary**

**What is already known on this topic?**

Leveraging mail and self-sampling can increase participation in colorectal cancer screening, but the range of approaches that use these strategies has not been widely investigated.

**What is added by this report?**

Across 8 research projects reaching diverse populations in the US, we observed multiple strategies that leverage mail and self-sampling to promote colorectal cancer screening.

**What are the implications for public health practice?**

Mail and self-sampling for colorectal cancer screening, including outreach to promote mailed fecal immunochemical tests, could be more broadly leveraged to optimize screening.

## Introduction

National guidelines, such as those from the US Preventive Services Task Force (USPSTF), recommend self-sampling methods for colorectal cancer (CRC) screening through guaiac fecal occult blood testing, and more recently, fecal immunochemical testing (FIT) and FIT-DNA testing (1,2). Self-sampling methods reduce structural barriers to cancer screening by removing the burden of visiting a health care site, and self-sampling, facilitated by mailed outreach, has been shown to increase CRC screening by an absolute 28% compared with usual, visit-based screening (3–5). Self-sampling methods are increasingly proposed for addressing inequities in screening and have been envisioned by the President’s Cancer Panel (6). In one integrated health system, mailed FIT outreach was a key component of a population health initiative that dramatically reduced CRC incidence (7) and eliminated disparities in CRC incidence and death between non-Hispanic Black and non-Hispanic White adults (8).

Despite its promise, mailed outreach using self-sampling is challenging to implement (9). Mailed outreach requires systems for mailing and processing samples, prompting patients to complete testing, communicating results to clinicians and patients, and ensuring timely follow-up of abnormal test results. Differences in how these systems are designed can influence how well a program is implemented and maintained and, ultimately, its effectiveness (10–13). However, many health system leaders and program planners lack knowledge about how to establish and adapt these systems for their context. In response to this knowledge gap, the Centers for Disease Control and Prevention (CDC) and the National Association of Chronic Disease Directors convened subject matter experts as part of a 2018 summit to identify optimal strategies for implementing mailed FIT outreach programs. Summit attendees identified several outreach components and practices that could lead to higher completion rates (hereinafter, Summit consensus recommendations) (9) and produced a mailed FIT implementation guide (14). The extent to which Summit consensus recommendations have been adapted and implemented has not been comprehensively characterized.

The National Cancer Institute’s (NCI’s) Accelerating Colorectal Cancer Screening and Follow-up through Implementation Science (ACCSIS) Consortium supports research to understand how evidence-based multilevel interventions, such as mailed FIT outreach, can be implemented and scaled to reduce the burden of CRC (15,16). Here, we present the range of approaches to promote mail-based self-sampling methods for CRC screening completion implemented by 8 ACCSIS research projects and the extent to which these approaches were consistent with Summit consensus recommendations for mailed FIT outreach. This study may help illuminate strategies for promoting and supporting successful implementation of mail-based strategies for increasing CRC screening and attenuating disparities across diverse contexts.

## Methods

This study was conducted as part of the NCI-funded ACCSIS Consortium. Its overall aim is to support transdisciplinary research at multiple sites to evaluate and improve CRC screening processes using implementation science. The Consortium is intended to provide an evidence base for multilevel interventions that increase rates of CRC screening, follow-up, and referral to care, and best practices for how multilevel interventions can be scaled up to reduce the burden of CRC in the US, particularly in groups with traditionally low rates of screening participation. The ACCSIS Consortium consists of 8 five-year research projects and a coordinating center. Research projects funded through the ACCSIS Cancer Moonshot Initiative include sites in California (San Diego), Illinois and Indiana (referred to as Chicago), Kentucky and Ohio (referred to as Appalachia), North Carolina, and Oregon. Three sites are supported through cancer center supplements and focus on American Indian populations in Arizona, New Mexico, and Oklahoma. Within each research project, interventions occur at multiple clinical care subsites that include settings such as tribal clinics and federally qualified health centers (FQHCs). Individual projects were funded based on site-specific proposals, and the Consortium is supported by a central coordinating center (RTI International). Individual sites are responsible for evaluating their own project performance and reporting performance via progress reports and peer-reviewed articles. Data sharing of common data elements pertinent to screening and follow-up is required, and plans to make these data available for the broad research community are required by NCI and are in process (17). ACCSIS evaluation plans include analysis of common data elements related to screening initiation and follow-up collected across all research projects. Additionally, the coordinating center works with sites to develop opportunities for trans-ACCSIS initiatives and analyses, such as the analysis provided here.

### The ACCSIS framework

The ACCSIS framework is a model for how to implement multilevel, evidence-based interventions to increase CRC screening, follow-up, and referral to care (Appendix). The framework identifies multilevel contextual factors that drive selection of evidence-based interventions, adaptations that can be made in response to local context, and an iterative process for implementing and evaluating the chosen interventions. The framework guides assessment of implementation success, as well as short-term and long-term outcomes (eg, increases in CRC screening and follow-up, reductions in CRC incidence and mortality). This framework provides overarching guidance for selecting the categories and data elements directly relevant to understanding how each ACCSIS research project proposes to leverage mail to promote CRC screening.

### Definitions of strategies for leveraging mail to promote completion of CRC screening

To facilitate consistent descriptions of each research project, we defined ways that mail could be used to promote screening completion. We intended our definitions to distinguish differences in 1) how someone is identified as needing a screening test, 2) how the test is delivered to the patient and returned for processing, and 3) which type of self-sampling test is offered. Our definitions intended to accommodate future innovations in test distribution and test type (eg, novel fecal test approaches, blood test using self-collection devices). For FIT, categories included organized mailed FIT outreach, organized on-demand mailed FIT, and opportunistic FIT with mailed return (Table 1). We created analogous definitions for FIT-DNA screening: organized FIT-DNA outreach, organized on-demand FIT-DNA, and opportunistic FIT-DNA. In so doing, we recognize that the only currently marketed FIT-DNA test (Cologuard) is not available for opportunistic, clinic-visit-based distribution of test kits with mailed return and that this test is not consistently available to all populations because of differences in insurance coverage and cost.

**Table 1:** Strategies for Leveraging Mail to Promote Colorectal Cancer Screening With Stool-Based Tests

Mail strategy	Definition and key components
Organized mailed FIT outreach	Organizational-level (eg, health system, health clinic, health insurance plan) or population-level identification of patients not up-to-date with screening for mailed outreach including a FIT kit with a postage-paid envelope to return the FIT to the laboratory via mail. May have a centralized component that crosses clinics within a system, individuals within a population, or health systems within a region. A common modification for clinic- and health system–based outreach is to specify or allow a patient to return a FIT to a laboratory or clinic by hand instead of through mail. Mailed FIT outreach is distinct from approaches that use FIT-DNA as the testing strategy.
Organized on-demand mailed FIT	Organizational-level or population-level outreach, via mailed letter, text message, or telephone call, to patients not up-to-date with screening to opt in to receive a FIT kit with a postage-paid return envelope. A common modification is for a patient to return a FIT to a laboratory or clinic by hand instead of through mail.
Opportunistic FIT with mailed return	Opportunistic in-person or virtual clinic–based or other health visit–based distribution of FIT to patients not up-to-date with screening with a postage-paid envelope to return FIT to laboratory. Opportunistic invitation could be based on in-visit invitation or review of scheduled patients not up-to-date with screening before a visit to prepare orders.
Organized FIT-DNA (Cologuard^a^) outreach	Organizational-level or population-level identification of patients not up-to-date with screening for mailed outreach including a FIT-DNA kit with instructions for completion and sample pickup by a mail courier.
Organized on-demand FIT-DNA	Organizational-level or population-level outreach via mailed letter, text message, or telephone call, to patients not up-to-date with screening to opt in to receive a FIT-DNA kit with instructions for completion and sample pickup.
Opportunistic FIT-DNA	Opportunistic in-person or virtual clinic–based or other health visit–based offer of a FIT-DNA kit to patients not up-to-date with screening with FIT-DNA order for laboratory to mail patient FIT-DNA kit with instructions for completion and sample pickup.

Abbreviation: FIT, fecal immunochemical test.

a Exact Sciences’ follow-up for ordered Cologuard includes outreach to explain testing process to patient, mailing kit to patient, and answering questions about test completion. Exact Sciences’ follow-up does not include reporting results to patient or coordination of colonoscopy for abnormal test results.

### Data collection

We created structured data collection instruments to summarize site and project characteristics based on the ACCSIS conceptual framework and Summit consensus–recommended approaches for mailed FIT outreach (9). The instrument followed the ACCSIS framework goal of characterizing contextual factors, intervention characteristics, and outcome metrics. Questions for the components of mailed FIT outreach followed the Summit consensus–recommended strategies (9,14).

For this article, “project lead” or “project champion” can refer to an individual, a group, or an institution. We iteratively reviewed and refined data through discussion and email communications from January 2021 to October 2022. Summarized data reflect initial implementation strategies used.

### Research ethics and regulations

Projects were approved by local institutional review boards. This article was reviewed and approved by each research project site; for American Indian sites, approval was based on local protocols for tribal leadership and Indian Health Service review.

## Results

All 8 ACCSIS research projects, representing rural, urban, and tribal settings in the South, Midwest, Southwest, and Northwest, participated in this study (Figure, Table 2, Table 3). Populations served by the research projects are racially and ethnically diverse, including American Indian, Black, Hispanic, Asian American, and non-Hispanic White populations, and individuals with lower socioeconomic position, who are more likely to be medically underserved. Seven projects (Appalachia, Arizona, Chicago, New Mexico, North Carolina, Oregon, and San Diego) initially focused on offering screening to individuals aged 50 to 75 years, whereas 1 project (Oklahoma) initially offered screening to individuals aged 45 to 75 years, consistent with the most recent USPSTF guidelines.

**Figure:**
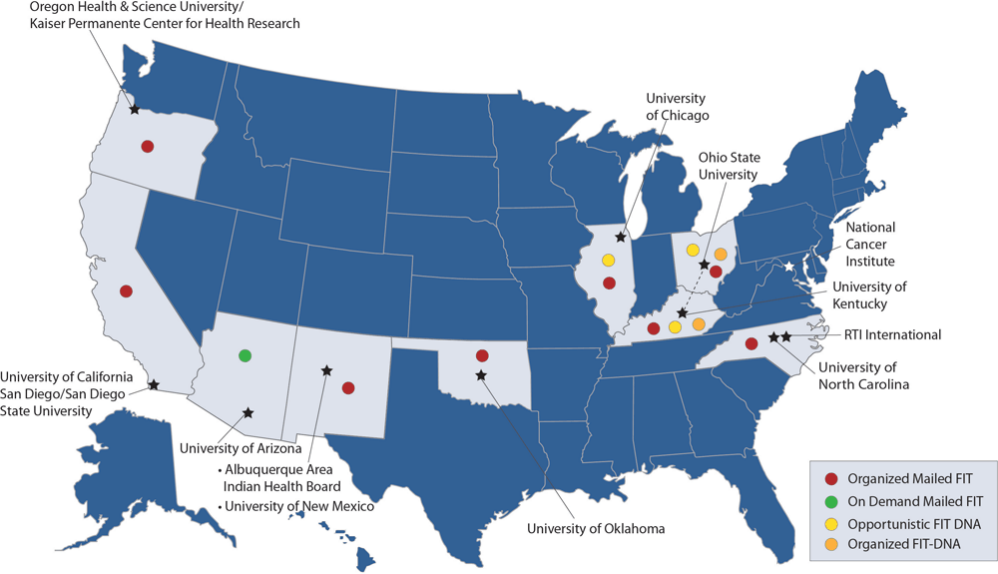
ACCSIS consortium members, research project sites, and mail-based strategies used for promoting CRC screening. Abbreviations: ACCSIS, Accelerating Colorectal Cancer Screening and Follow-up through Implementation Science; CRC, colorectal cancer; FIT, fecal immunochemical test.

**Table 2:** Characteristics of 8 ACCSIS Sites and Primary Strategies for Leveraging Mail for CRC Screening Completion

ACCSIS site	Characteristic
**Region served**	
Appalachia	12 Appalachian counties in Ohio and Kentucky
Arizona	American Indian communities served by an FQHC or 1 of 2 rural PL 93–638 (Indian Self-Determination And Education Assistance Act of 1975) facilities in Arizona
Chicago	Chicago, Illinois, and Indiana
New Mexico	Largely rural American Indian communities in the Albuquerque Area Southwest Tribal Epidemiology Center Service Area (New Mexico and Texas)
North Carolina	Regions in North Carolina with high CRC burden and low CRC screening
Oklahoma	3 Sites: 2 rural and 1 in an urban center
Oregon	Rural and frontier communities of Oregon
San Diego	San Diego County, California
**Priority population and type of health system or clinic**	
Appalachia	Ages 50–74, rural and medically underserved
Arizona	American Indians aged 45–75 years at average risk for CRC without exclusions
Chicago	Ages 50–74, racial and ethnic minority and low-income populations
New Mexico	American Indians aged 45–75 years at average risk for CRC served by tribally operated health care facilities
North Carolina	Ages 50–74, not up-to-date, served by 1 of 2 FQHC systems
Oklahoma	American Indians aged 45–75 years at average risk for CRC served by 1 tribally operated health care group (8 clinics), 1 Indian Health Service–affiliated tribal clinic, and 1 urban clinic
Oregon	Ages 50–74, Medicaid and dual (Medicaid–Medicare) recipients in 28 clinics served by 3 Medicaid health plans
San Diego	Ages 50–75 years, not up-to-date, served by 1 of 3 FQHC systems
**Overall approach**	
Appalachia^a^	Strategy A, Opportunistic FIT-DNA and FIT with mailed return and organized mailed FIT (1 clinic in Ohio); see Table 3 for additional strategies used
Arizona	On-demand mailed FIT
Chicago^b^	Organized mailed FIT outreach (12 clinics within 1 FQHC health system in Indiana); see Table 3 for additional strategies used
New Mexico	Organized mailed FIT outreach
North Carolina	Organized mailed FIT outreach
Oklahoma	Organized mailed FIT outreach
Oregon	Organized mailed FIT outreach
San Diego	Organized mailed FIT outreach
**Project leader**	
Appalachia	FQHC clinic system
Arizona	Physician leader at tribal health clinic or FQHC supervising full-time patient navigator
Chicago	FQHC clinic system
New Mexico	Tribal health clinic
North Carolina	FQHC clinic system champions in collaboration with a centralized screening outreach entity at an academic cancer center
Oklahoma	Patient navigator at each tribal clinic location
Oregon	Medicaid Health plans (CCOs) in collaboration with clinics in their network and contracted third-party vendors
San Diego	Nonprofit organization that partners with FQHCs to improve health outcomes with the nonprofit helping to deliver mailed outreach to FQHC patients
**Patient identification**	
Appalachia	EHR data queries
Arizona	Identified by reviewing lists of patients scheduled for upcoming clinic visits; patients not attending an upcoming clinic visit were called to be offered a FIT to be sent by mail
Chicago	Generated list from population health management tools
New Mexico	EHR query followed by manual review by a nurse to confirm eligibility (“scrubbing”)
North Carolina	Query of EHR data, adapted to EHR vendor
Oklahoma	iCare list (a software tool used to assist with patient management) updated each month with new patients as they become eligible, information from EHR queries
Oregon	List generated by CCOs based on claims data and reviewed by clinic staff using EHR data (ie, scrubbed)
San Diego	EHR data queries, which are then scrubbed by FQHC partners
**Outreach components**	
Appalachia	Letter, CDC’s “Screen for Life” CRC screening fact sheet, low-literacy instructions
Arizona	Cover letter, CRC education, FIT instructions, FIT return mailer packet, postage included
Chicago	Letter and FIT kits, patient reminders using text messages with hyperlinks to educational written materials and videos, reminder telephone call by patient navigator if FIT not completed within 60 days
New Mexico	Letter, brochure, FIT kit, instructions
North Carolina	Primer letter, followed by mailed FIT packet with education pamphlet, FIT instructions, FIT kit, and return mailer packet with postage included, and 2 mailed reminders
Oklahoma	Letter, brochure, FIT kit, telephone calls, and instructions, as well as telephone and mail follow-up with patients who do not return the kit
Oregon	Primer/alert letters sent by vendors; FIT mailing with FIT, instructions, and cover letter; variable reminders following the mailing, including vendor-supported mailed letters, text reminders, or auto calls; clinic staff could make live telephone calls
San Diego	Invitation letter including a mailed FIT, mailed/text primers, automated call/text reminders
**Test return**	
Appalachia	Stamped addressed return envelope sent to FIT-DNA manufacturer (Exact Sciences) or clinical laboratory
Arizona	Clinic drop-off or pickup by patient navigator
Chicago	Mailed back to the clinic
New Mexico	Patients hand-carry FIT kits to the clinic or have them picked up at the home by a navigator
North Carolina	Mail to commercial laboratory
Oklahoma	Mail to clinic laboratory or clinic drop-off
Oregon	Mailed back to clinics, central laboratories, or vendor; drop off at the clinic
San Diego	By mail to either FQHC on-site laboratories or a commercial reference laboratory
**Outreach delivery**	
Appalachia	Exact Sciences mails FIT-DNA kit to patients after referral is made by clinic staff. FITs are mailed by health center staff
Arizona	Clinic-based navigator
Chicago	FIT program team at FQHC mails FIT kits to patients
New Mexico	Navigator or community health worker in clinic and community-based settings (eg, health fairs) based at the tribal health clinic, in-person outreach, newsletters, and FIT kit mailings
North Carolina	Outreach center based at academic cancer center
Oklahoma	Patient navigator initiated from the tribal clinic
Oregon	Third-party vendor for each CCO supports alerts, mailing, and reminders. Clinic staff are encouraged to make live telephone calls to support FIT mailing
San Diego	Third-party vendor
**Test result follow-up**	
Appalachia	Health center staff shares all test results (normal and abnormal) with the patient by telephone
Arizona	Normal and abnormal test results delivered by the clinic-based navigator via telephone. For abnormal test results, each clinic’s study navigator tracks and leads patients through the process of completing a diagnostic colonoscopy.
Chicago	Follow-ups for abnormal test results by a patient navigator at the FQHC system
New Mexico	For normal test results, clinic sends a letter with results. For abnormal test results, a public health nurse based at the clinic provides navigation for diagnostic colonoscopy.
North Carolina	For normal test results, a letter is sent. Abnormal test results are communicated to patients through usual care processes, and followed with care coordination per usual care, as well as centralized telephone-based navigation by the outreach team
Oklahoma	Telephone call or letter with an informative pamphlet sent to the patient by clinic-based patient navigator
Oregon	For normal test results, patients received either a letter or telephone call from the clinic or the vendor (workflow tailored to setting). For abnormal test results, patients receive a telephone call from the provider/care team or from a central care management team (1 CCO). Patient navigators from the clinics follow up with all abnormal FIT results to support colonoscopy completion.
San Diego	Individuals with abnormal FIT results are tracked and navigated at the clinic level by using a project-specific protocol. Individuals with normal FIT results receive letter as well as usual care clinic processes.
**Primary outcome**	
Appalachia	Screening completion rate
Arizona	Increase screening completion rate by ≥25% over baseline
Chicago	Screening completion rates, time from mailing to returning, and for whom it is effective (ie, identify moderators)
New Mexico	Proportion returning FIT
North Carolina	Screening completion at 6 months in usual care vs mailed FIT intervention arm
Oklahoma	Proportion returning FIT and the proportion of abnormal FIT results
Oregon	Differences between intervention and usual care groups in receipt of any CRC screening within 6 months of patient identification
San Diego	Improvement in CRC screening rates over 3 years, comparing intervention vs nonintervention clinics

Abbreviations: ACCSIS, Accelerating Colorectal Cancer Screening and Follow-up through Implementation Science; CCO, Coordinated Care Organization; CDC, Centers for Disease Control and Prevention; CRC, colorectal cancer; EHR, electronic health record; FIT, fecal immunochemical test; FQHC, federally qualified health center.

a Appalachia tested 5 additional strategies (Table 3).

b Chicago tested 1 additional strategy (Table 3).

**Table 3:** Characteristics of and Additional Strategies Used by Appalachia and Chicago ACCSIS Sites for Leveraging Mail for CRC Screening Completion

Characteristic	Appalachia				Chicago
	Strategy B	Strategy C	Strategy D	Strategy E	
Location	6 clinics in Kentucky	1 clinic in Ohio	1 clinic in Ohio	2 clinics in Ohio	13 clinics within 1 FQHC system in Chicago
Overall approach	Opportunistic FIT-DNA	Opportunistic FIT with mailed return and organized on-demand mailed FIT	Opportunistic FIT with mailed return and organized on-demand mailed FIT	Opportunistic FIT-DNA and FIT with mailed return and organized FIT-DNA outreach	Opportunistic FIT-DNA
Project leader	FQHC clinic system or hospital-based rural health clinic	FQHC clinic system	FQHC clinic system	FQHC clinic system	FQHC system
Patient identification	EHR data queries	EHR data queries	EHR data queries	EHR data queries	EHR queries
Outreach components	Letter, test kit, instructions, QR code link to video instructions	Letter, CDC “Screen for Life” CRC screening fact sheet, low-literacy instructions	Letter, CDC “Screen for Life” CRC screening fact sheet, low-literacy instructions	None (all provided by Exact Sciences)	FIT-DNA kits mailed to patients by Exact Science
Test return	Prepaid return box addressed to FIT-DNA manufacturer (Exact Sciences) for pickup at home by courier or drop-off at clinic for subsequent courier pickup	Stamped addressed return envelope sent to commercial laboratory	Stamped addressed return envelope sent to commercial laboratory or picked up from patient by caseworker and provided to commercial laboratory by health center staff	Stamped addressed return envelope sent to commercial laboratory	FIT-DNA kits are sent to Exact Sciences
Outreach delivery	Exact Sciences mails FIT-DNA kit to patients after referral by clinic staff	FITs mailed by health center staff	Mailed by health center staff	Exact Sciences mails FIT-DNA kit to patients after referral is made by clinic staff, FITs mailed by health center staff	Telephone calls and text reminders
Test result follow-up	Health center staff shares all results with the patient by telephone	Health center nurse or provider shares all abnormal test results with patient by telephone; nurse or provider shares normal test results with patient by mail	Health center nurse or provider shares all results with patient by telephone	Health center nurse or provider shares all results with patient by telephone	Follow-up for abnormal test results by patient navigator at the FQHC system
Primary outcome	Screening rate	Screening rate	Screening rate	Screening rate	Screening completion rates, time from mailing to returning, and for whom it is effective (ie, identify moderators)

Abbreviations: ACCSIS, Accelerating Colorectal Cancer Screening and Follow-up through Implementation Science; CDC, Centers for Disease Control and Prevention; CRC, colorectal cancer; EHR, electronic health record; FIT, fecal immunochemical test; FQHC, federally qualified health center.

### Approach to leveraging mail for outreach

All 8 ACCSIS research projects reported leveraging mail to facilitate completion of stool-based CRC screening, with multiple approaches represented (Figure). Seven of 8 projects used organized mailed FIT outreach (Appalachia, Chicago, New Mexico, North Carolina, Oklahoma, Oregon, and San Diego). Additionally, 1 project used on-demand mailed FIT (Arizona), 2 projects used opportunistic FIT-DNA (Appalachia and Chicago), and 1 project used organized FIT-DNA (Appalachia). Four research projects reported a plan to enhance opportunistic FIT (Appalachia, Arizona, Chicago, and New Mexico), and many other projects reported that opportunistic FIT was operational in usual care.

**Project leads.** Project leads included a nonprofit organization that supports FQHC health systems for 1 project (San Diego), 3 Medicaid health plans and affiliated clinics for 1 project (Oregon), 1 or more FQHC health systems for 3 projects (Appalachia, Arizona, and Chicago), a coalition of an FQHC health system and an academic cancer center for 1 project (North Carolina), and tribal health clinics for 3 projects (Arizona, New Mexico, and Oklahoma (Table 2).

**Patient identification strategies.** Diverse approaches were used for patient identification, including electronic health record queries (n = 7; Appalachia, Arizona, Chicago, New Mexico, North Carolina, Oklahoma, and San Diego), insurance claims data (n = 1; Oregon), electronic population health tools (n = 3; Chicago, Oklahoma, and San Diego), and upcoming patient appointment lists (n = 1; Arizona) (Table 2 and Table 3). Some research projects described additional “scrubbing” procedures in which initial data queries were reviewed to verify accuracy (n = 4; New Mexico, North Carolina, Oregon, and San Diego). The number of patients receiving mailed FIT outreach differed by research project and ranged from 180 to thousands of patients per year.

**Mailed components.** At a minimum, mailed components included an opportunity for self-collection and mailed return of a FIT or FIT-DNA kit (Table 2 and Table 3). For sites using opportunistic FIT-DNA (Appalachia and Chicago) and organized FIT-DNA (Appalachia), the mailed components were supplied by the manufacturer and included an invitation to complete screening, a FIT-DNA kit, and instructions on how to complete the kit and arrange for return to the FIT-DNA laboratory.

**Outreach delivery and test return approaches.** Outreach was delivered by a health system or clinic (Appalachia, Arizona, and Chicago), an academic cancer center (North Carolina), a third-party mail fulfilment service (Oregon and San Diego), and a tribal health clinic (Arizona, New Mexico, and Oklahoma). Stool test return strategies varied across research projects, and many offered more than 1 option: mailed return to a health clinic laboratory or commercial laboratory (n = 6; Appalachia, Chicago, North Carolina, Oklahoma, Oregon, and San Diego), home pickup by a case worker or patient navigator (n = 3; Appalachia, Arizona, and New Mexico), and in-person return to a health clinic laboratory (n = 5; Appalachia, Arizona, New Mexico, Oklahoma, and Oregon).

**Follow-up on abnormal test results.** Seven projects (Appalachia, Arizona, Chicago, New Mexico, Oklahoma, Oregon, and San Diego) offered clinic-based navigation and care coordination, and 1 project used a combination of clinic-based care coordination (usual care) plus central telephone navigation (North Carolina).

**Primary project outcome.** For all research projects, the primary outcome was screening completion by any USPSTF guideline–recommended modality, including stool testing or colonoscopy. The time frame for assessing primary outcome ranged from completion within 6 months to within 12 months for most projects; 1 project assessed change in proportion up-to-date with screening at the health system from baseline through 3 years follow-up.

### Use of Summit consensus recommendations for mailed FIT outreach across ACCSIS sites

Seven of 8 projects (all but Arizona) reported using organized mailed FIT outreach to promote screening (Table 4A and Table 4B). However, the scale of outreach varied on the basis of factors such as clinic preferences, clinic staffing, and availability of patients with home mailboxes. For example, some projects initiated batch mailings to hundreds of patients in each mailing cycle, leveraging electronic health records (North Carolina, Oklahoma, and San Diego) or claims data (Oregon), and 1 project reported that clinics mail 10 to 30 kits per month depending on staff availability (Appalachia). Project sites used all or nearly all 8 Summit consensus–recommended strategies.

**Table 4A:** Use of 8 Summit Consensus Recommendations for Mailed FIT Outreach Across 7 ACCSIS Projects That Used Mailed FIT Outreach^a^^,^ b

Recommended strategy	Arizona	Appalachia	Chicago	New Mexico
**Use primers, such as texts, telephone calls, and printed mailings, before mailed outreach**				
Strategy used?	No	Yes	Yes	Yes
Approach	Primer in English and native language is mailed to the minority of clients who have a home postal address. Invitations are not sent to post office boxes.	1-Page primer flyer sent 2 weeks before mailing.	Initial live call to let the patient know they are not up to date and to expect a kit in the mail and provide patient education on CRC screening.	Culturally tailored printed materials for mass distribution in community newsletter, brochures, and flyers and in clinic- or community-based settings. Materials were also disseminated at checkpoints (during COVID-19) and at health fairs. These were not mailed except for the community newsletter.
**Use a brief, easy-to-read invitation letter, with signatory tailored to setting**				
Strategy used?	Yes	Yes	Yes	Yes
Approach	Brief invitation on clinic letterhead.	1-Page invitation letter in English outlines importance of CRC screening, basics of the at-home test, and instructions for returning the test. Letter is signed by health care provider or general care team.	Invitation letter signed by the health system.	Invitation letter on clinic letterhead and signed by the clinic director was sent with the mailing.
**Use simple FIT completion instructions that address challenges such as failed laboratory processing, literacy, and language**				
Strategy used?	Yes	Yes	Yes	Yes
Approach	Manufacturers’ instructions included with kits with cover letter providing instructions for return of kits, in English and tribal language.	Mixed text and simple pictorial instructions (in English) for proper test completion and sample mailing.	Simple written, bilingual, and pictorial instruction. Also, hyperlinks to instruction videos through text message.	Used manufacturer’s instructions and an additional instruction sheet (mixed pictorial and text) for mailed FIT, all in English. Some included a QR code linking to an instructional video.
**Use a high-quality, 1-sample FIT**				
Strategy used?	Yes and no	Yes and no	Yes and no	Yes and no
Approach	OC-Light S, Hemoccult ICT, and Hemosure at 1 facility each.	Will use whichever test is used by the health system (ie, OC-Auto FIT, Hemosure, FIT-DNA)	InsureONE	OC-Auto and InsureONE based on tribally operated health care facilities’ usual-care FIT.
**Use reminders to initial noncompleters to increase the return rate (mailed, telephone, text, email, text-video)**				
Strategy used?	Yes	Yes	Yes	No
Approach	Live telephone follow-up twice by navigator, leaving voice/text message if necessary.	Live telephone follow-up.	Patient reminder using text message, live telephone reminder from a patient navigator.	Planned but not implemented because of COVID-19 disruptions. Staff were repurposed for COVID-19 mitigation efforts throughout the health care facility.
**Establish a data infrastructure to identify eligible patients and track each step in outreach process**				
Strategy used?	Yes	Yes	Yes	Yes
Approach	Eligible patients scheduled for clinic visits are identified through EHRs. These patients are entered by navigators into a project-specific REDCap database to track completion of all further steps in screening process.	Patients are identified by EHR data queries and morning huddle reports for clinic visits; progress through screening process is tracked via Excel spreadsheets and limited EHR use.	Collect quarterly data from EHR to track completion, results, and follow-up colonoscopy if needed. Data are extracted from EHR in an Excel format.	Implemented EHR enhancements and coded for historical colonoscopies to facilitate EHR functionality to identify eligible patients. A project-specific database was used to track mailed FIT kits, completion, and reminders. The project-specific tool was a written log, EHR-based tool, or Excel spreadsheet.
**Use protocols and procedures such as navigation to promote colonoscopy after abnormal FIT**				
Strategy used?	Yes	Yes	Yes	Yes
Approach	Monthly videoconference with navigators to track aggregate results, including abnormal FIT results and follow-up thereof.	No formal patient navigator, but follow-up plan will be specific to individual health centers. Patients with abnormal at-home test results are navigated by clinic staff to resolution (via colonoscopy referral and completion) according to the clinic’s written CRC screening and follow-up care plan and pathway.	Patient navigator will follow up with patients who have an abnormal FIT result and help them obtain a diagnostic colonoscopy.	Clinic providers deliver consultation and referral to colonoscopy with an offer of patient navigation. Public health nurses navigate patients to diagnostic follow-up. The purchase/referral care program also makes referrals and navigates patients for diagnostic follow-up.
**Identify a project champion and organizational support to promote sustainability**				
Strategy used?	Yes	Yes	Yes	Yes
Approach	Project leaders and physicians stationed at clinic facilities serve as project champions. Sustainability discussions are ongoing with project champions and the study team, as well as the medical directors of each clinic.	A clinic champion was identified and trained as a project champion for each individual health center, including to promote sustainability.	Mailed FIT team at health system.	The project champions, who are members of the multisector action teams, support enhancement of CRC. The multisector action team members are health care providers and represent various sectors in the tribal health clinics. Project champions are engaged in discussions on sustainability, which will be based on the effectiveness of various implementation strategies being implemented/tested.

Abbreviations: ACCSIS, Accelerating Colorectal Cancer Screening and Follow-up through Implementation Science; CDC, Centers for Disease Control and Prevention; CRC, colorectal cancer; EHR, electronic health record; FIT, fecal immunochemical test; FQHC, federally qualified health center.

a The Centers for Disease Control and Prevention and the National Association of Chronic Disease Directors convened subject matter experts as part of a 2018 summit to identify optimal strategies for implementing mailed FIT outreach programs. Summit attendees identified 8 outreach components and practices that could lead to higher completion rates (9).

b Arizona did not use mailed FIT outreach, but as part of on-demand mailed FIT, the project used many components often included as part of mailed FIT outreach; these are shown for comparison.

**Table 4B:** Use of 8 Summit Consensus Recommendations for Mailed FIT Outreach Across 7 ACCSIS Projects That Used Mailed FIT Outreach^a^^,^ b

Recommended strategy	North Carolina	Oklahoma	Oregon	San Diego
**Use primers, such as texts, telephone calls, and printed mailings, before mailed outreach**				
Strategy used?	Yes	Yes	Yes	Yes
Approach	Printed mailed primer letters (English and Spanish) noting that a follow-up mailing will include a FIT kit.	Telephone calls (both live and automated) and mailed information.	Selected based on CCO/vendor agreements, and included bilingual introduction letter sent 1 week before mailing (2 CCOs). Clinics encouraged to supplement with a telephone call for patients at risk for nonresponse (eg, no prior CRC screening, newly age eligible).	Mailed primer (English/Spanish) describing the importance of CRC screening and noting that follow-up mail will include a FIT kit. Text message primer alerting that a FIT kit is on the way.
**Use a brief, easy-to-read invitation letter, with signatory tailored to setting**				
Strategy used?	Yes	Yes	Yes	Yes
Approach	1-Page invitation letter (English/Spanish) outlines reason for invitation; goal of project; and option to opt out of the project. Letter is signed by FQHC site medical director.	Letters signed by patient navigator to patients eligible for screening.	For 2 CCOs, invitation letters were on clinic letterhead and signed by the clinic care team. For 1 CCO, the invitation letter was on the CCO letterhead and signed by the CCO.	The 1-page invitation letter (English/Spanish) outlines the reason for the invitation, goal of the project, and option to opt out. The letter is signed by the clinic health system.
**Use simple FIT completion instructions that address challenges such as failed laboratory processing, literacy, and language**				
Strategy used?	Yes	Yes	Yes	Yes
Approach	Pictorial instructions with minimal text (English/Spanish) targeted for low literacy, emphasizing importance of writing collection date on sample collection device and sending test promptly.	Manufacturers’ instructions included with kits with cover letter providing instructions for their return.	2 CCOs used simple, pictorial FIT instructions. (mailedfit.org). 1 CCO used instructions that came with the FIT from the manufacturer because of the timeline for compliance/legal team review.	Mixed pictorial and text instructions (English/Spanish) targeted for low literacy, modified from manufacturer instructions, emphasizing importance of writing collection date on sample collection device and sending test promptly.
**Utilize a high-quality, 1-sample FIT**				
Strategy used?	Yes and No	Yes and No	Yes and No	Yes and No
Approach	OC-Auto for mailed FIT intervention. UC FIT varied by site.	InsureONE, OC-Light S, and Hemosure FIT.	FIT selection was aligned with clinic/CCO/vendor. Most clinics are using high-quality 1-sample FITs: OC-Auto (Polymedco), OC-Light S (Polymedco); InSure ONE (Clinical Genomics); and Hemosure iFOB Test Kit (Hemosure, Inc). 1 clinic is using 2-sample OneStep+ (Henry Schein, Inc). When vendors allowed, some clinics chose their own FITs to accommodate laboratory processing, staff knowledge, and clinic workflows.	OC-Auto and InsureONE, based on patient’s insurance or health system’s usual-care FIT.
**Use reminders to initial noncompleters to increase the return rate (mailed, telephone, text, email, text-video)**				
Strategy used?	Yes	Yes	Yes	Yes
Approach	2 Mailed, printed reminders 2 weeks apart for patients who did not return FIT.	Live telephone reminders.	1 CCO sent text message reminders 1 week after FIT mailing and 2 weeks later. 1 CCO sent a mailed reminder letter. All clinics were encouraged to make live reminder telephone calls 1–2 weeks after FIT mailing. Many of those calls happened much later or not at all because of staffing disruptions.	For patients who did not return FIT within 2 weeks of invitation, a third-party vendor delivered reminders via automated calls and text messages.
**Establish a data infrastructure to identify eligible patients and track each step in outreach process**				
Strategy used?	Yes	Yes	Yes	Yes
Approach	Eligible patients identified by EHR queries specific to each site’s EHR vendor and scrubbing (at 1 site). A secure REDCap database was used for tracking mailed FIT intervention process steps (eg, bad address, delivery of reminders).	Screen-eligible patients are identified from the EHR at each partnering facility. Eligible patients were entered by navigators into a project-specific Excel worksheet to track completion of all further steps in screening process. Data are collected via an SQL program that extracts nonconfidential data from the Excel spreadsheet.	CCOs extracted the list of eligible patients from their claims database. The list is reviewed and cleaned by the research team, then uploaded to a REDCap system for all subsequent steps related to intervention tracking and delivery (eg, scrubbing, mailing, reminder calls, FIT result, navigation initiated).	For identification of eligible patients and also results of returned FIT, a data query system that aggregates quality metric data from across FQHC systems (Arcadia) was used. 1 System scrubbed the list of eligible patients found through the initial query. For tracking mailed FIT intervention process steps (eg, bad address, delivery of reminders), a third-party vendor provided a web-based query interface.
**Use protocols and procedures such as navigation to promote colonoscopy after abnormal FIT**				
Strategy used?	Yes	Yes	Yes	Yes
Approach	Primary care physicians notified of abnormal FIT result via EHR message. All patients with an abnormal test result are offered telephone navigation from a centralized outreach center at cancer center. Navigator followed a barriers-based navigation protocol and had access to financial and transportation assistance resources.	Patient navigators facilitate referral for colonoscopy for all abnormal FIT kit results and deliver letters to patients with abnormal test results.	The research team has trained designated clinic staff in patient navigation. Patient navigation is supported and documented in the REDCap tracking system.	Community health clinic care coordinators were trained on a specific protocol for promoting abnormal FIT results follow-up, including a checklist for monitoring key steps, and strategies for addressing barriers.
**Identify a project champion and organizational support to promote sustainability**				
Strategy used?	Yes	Yes	Yes	Yes
Approach	The project champion is the implementation research team at the cancer center organization, with buy-in from medical directors and quality-improvement champions at each site with regular participation in meetings. The cancer center has initiated conversations with other partners to develop policy and funding strategies for sustaining mailed outreach beyond the grant funding period.	Mailed FIT team at health system.	Each participating CCO and clinic site identified a primary point of contact and implementation team. These leads helped support the decision to take part in the research study and subsequent implementation and evaluation activities. Who filled these roles varied by practice site and structure.	The project champion is a central quality promotion organization. The organization has initiated conversations with other partners to develop policy and funding strategies for sustaining mailed outreach beyond the grant funding period.

Abbreviations: CCO, coordinated care organizations; CRC, colorectal cancer; EHR, electronic health record; FIT, fecal immunochemical test; FQHC, federally qualified health center; SMS, short messaging service.

a The Centers for Disease Control and Prevention and the National Association of Chronic Disease Directors convened subject matter experts as part of a 2018 summit to identify optimal strategies for implementing mailed FIT outreach programs. Summit attendees identified 8 outreach components and practices that could lead to higher completion rates (9).

b Arizona did not use mailed FIT outreach, but as part of on-demand mailed FIT, the project used many components often included as part of mailed FIT outreach; these are shown for comparison.

**Use primers such as texts, telephone calls, and printed mailings before mailed outreach**. All 7 projects reported use of primers before sending a FIT by mail; however, mode of primers varied, with 6 projects reporting use of printed material (Appalachia, New Mexico, North Carolina, Oklahoma, Oregon, and San Diego), 1 project delivering a text primer (San Diego), and 3 projects reporting use of live telephone call primers in all (Chicago and Oklahoma) or some (Oregon) clinics.

**Use a brief, easy-to-read invitation letter with signatory tailored to setting.** All 7 projects reported efforts to use a brief, easy-to-read letter. Signatories were tailored to setting and varied from clinic directors (n = 2; New Mexico and North Carolina), to a health system or health plan (n = 3; Chicago, Oregon, and San Diego), to a health care provider or health care team (n = 2; Appalachia and Oregon), to a patient navigator (n = 1; Oklahoma).

**Use simple FIT completion instructions that address challenges such as failed laboratory processing, literacy, and language.** The range of FIT completion instruction types included pictorial (n = 1; Oregon), mixed pictorial and text (n = 5; Appalachia, Chicago, New Mexico, North Carolina, and San Diego), links to instructional videos (n = 1; Chicago), and manufacturer instructions (n = 3; New Mexico, Oklahoma, and Oregon). Notably, projects reporting use of pictorial, mixed pictorial and text, and instructional videos all noted efforts to optimize instructions for literacy level, and 5 projects offered instructions in a language other than English (Arizona, Chicago, North Carolina, Oregon, and San Diego).

**Use a high-quality, 1-sample FIT.** All 7 projects reported use of a 1-sample FIT kit for at least 1 clinic site. Six (Appalachia, New Mexico, North Carolina, Oklahoma, Oregon, and San Diego) used OC-Auto and OC-Light S (Polymedco), 5 projects (Chicago, New Mexico, Oklahoma, Oregon, San Diego) used InSure ONE (Clinical Genomics), and 3 projects (Appalachia, Oklahoma, Oregon) used Hemosure iFOB Test Kit (Hemosure, Inc). Additionally, in 1 project (Oregon), a site elected to use the 2-sample OneStep+ (Henry Schein, Inc).

**Use reminders to initial noncompleters to increase return rate.** Seven projects reported use of reminders to promote screening completion, including text messages (n = 3; Chicago, Oregon, and San Diego), live telephone calls (n = 4; Appalachia, Chicago, Oklahoma, and Oregon), automated telephone calls (n = 3; San Diego, Oklahoma, and Oregon), and mailed letters (n = 2; North Carolina and Oregon). One site (New Mexico) had planned to use telephone call reminders but was unable to because of staffing shortages precipitated by the COVID-19 pandemic.

**Establish a data infrastructure to identify eligible patients and track each step in the outreach process.** All projects reported establishing a data infrastructure to identify eligible patients and track each outreach step. These ranged from using an electronic health record tool (n = 4; Chicago, New Mexico, Oklahoma, and San Diego), to electronic spreadsheets (n = 3; Appalachia, New Mexico, and Oklahoma), to a web-based tool from a third-party vendor (n = 3; San Diego, North Carolina, and Oregon).

**Use protocols and procedures such as navigation to promote colonoscopy completion after abnormal FIT results.** All projects applied protocols and procedures to promote colonoscopy after an abnormal FIT result.

**Identify a project champion and organizational support to promote sustainability.** The project champions varied across projects and included primary point of contact for project activities (n = 1; Oregon), clinic-based quality improvement teams (n = 5; Appalachia, Chicago, New Mexico, Oklahoma, and Oregon), a centralized quality improvement team working across multiple health systems (n = 1; San Diego), and an NCI-designated cancer center (n = 1; North Carolina). Five projects confirmed discussions about organizational support to promote sustainability of the project (Appalachia, New Mexico, North Carolina, Oregon, and San Diego).

During the process of data collection, synthesis, and discussion, several challenges to mailed FIT outreach were reported, including limited staff time to prepare and send invitations at some clinics (a challenge that was exacerbated by COVID-19); data aggregation, such as for identifying individuals for mailed FIT outreach; and navigating how to use multiple different FIT kit brands on the basis of insurance plan or clinic selection.

## Discussion

Self-sampling methods combined with convenience of mail for test distribution and/or return have great potential for optimizing participation in cancer screening, including CRC screening. Among 8 ACCSIS research projects, various mail-based approaches were used, with some projects distributing tests by mail, offering mailed return of completed tests, or both. For research projects that delivered organized mail-based FIT distribution and return, nearly all Summit consensus–recommended best practices for mailed FIT outreach were implemented, suggesting that implementation is feasible across a range of geographic regions and populations. Nevertheless, we observed variation in how research projects designed and adapted outreach to address the unique needs of settings and populations. Our results have implications for using mail and self-sampling to promote the completion of screening for various cancers and offer a guide to how self-sampling tests, such as FIT, can be successfully implemented in diverse settings and populations.

Specific to organized mailed FIT outreach, our observations add to the growing body of evidence supporting its viability as a strategy for promoting CRC screening across diverse settings and populations. Seven of 8 research projects reported using organized mailed FIT outreach, with consistent use of Summit consensus–recommended best practices, albeit with differences in scale and scope, form of components, and site-specific challenges. For example, 7 projects delivered reminders to increase return rates, and these reminders were in various forms: letters, live telephone calls, automated calls, and text messages. All 7 of these research projects reported using a 1-sample FIT, and 1 project (Oregon) also used a 2-sample FIT at 1 clinic. Applying findings from an evidence synthesis on FIT performance commissioned by the Agency for Healthcare Research and Quality, which concluded that adequate evidence supports the performance of OC-Auto and OC-Light (18) but not of Hemosure or InsureONE (18), we conclude that 7 ACCSIS research projects offered a high-quality 1-sample FIT in at least 1 clinic or population. All projects reported using a data infrastructure to track steps in the outreach process, which facilitated monitoring of FIT return and completion of follow-up colonoscopy and could be leveraged for detecting and managing implementation issues.

Context shaped mailed FIT outreach delivery. For example, in Oregon, the ability to engage with rural Medicaid-managed care providers and challenges in producing data to support CRC outreach at rural clinics led to a mailed FIT outreach project driven by insurance claims data and facilitated by Medicaid health plans (19). In San Diego, the presence of a nonprofit organization whose mission is to optimize health care quality across multiple FQHC systems allowed for a mailed FIT outreach project led by this centralized entity. Potential for disparities in access to an optimal mailed FIT outreach was reflected in 3 observations: 1) some lower-resource clinics had limited capacity to scale mailed outreach beyond a handful of patients per month, 2) a lack of personal US mailboxes limited access to this mailed approach for some tribal communities, and 3) the brand of FIT was driven by insurance and other factors rather than by test quality alone (20). As such, even though mailed FIT outreach has been shown to be effective for increasing screening, its reach still may be limited without large-scale structural changes (eg, funding for national, statewide, or regional mailed outreach programs; addressing postal access for tribal communities). Despite these challenges, our observations suggest that multiple approaches can be taken to successfully implement best practices for mailed FIT outreach and that flexible approaches may be needed to meet the needs of diverse populations and settings.

Our findings also illustrate areas where additional research may be warranted. Although abundant evidence supports use of mailed FIT outreach and opportunistic mailed FIT, less research supports the use of organized on-demand mailed FIT. Across types of mail-based strategies, few head-to-head comparisons have been made of opportunistic, organized on-demand, and organized outreach for promoting FIT completion. In a direct comparison of organized mailed FIT outreach and organized on-demand FIT among Medicaid beneficiaries, Brenner et al found that rates of screening completion were higher with organized mailed FIT outreach (21). No studies have reported on the success of organized outreach offering FIT compared with organized outreach offering FIT-DNA or on-demand compared with opportunistic FIT. Understanding relative effectiveness of these approaches can optimize self-sample–based cancer screening for CRC and other at-home screening or diagnostic tests, such as human papillomavirus–DNA tests, or at-home blood collection for various tests (where small quantities of blood are required). Our Summit consensus–based definitions for mail-based self-sampling strategies may facilitate more consistent reporting on evaluations of mail-based screening strategies and better ability to systematically compare these approaches.

### Limitations

A few limitations may be considered in interpreting this report. ACCSIS research projects are not representative of all regions and populations in the US, although all projects attempted to conduct their studies as pragmatic studies. Implementation time frames included the start of the COVID-19 pandemic. As such, observations reported here might have been different if the pandemic had not occurred, but they do reflect the likely reality of a context in which COVID-19 remains active and at least endemic.

### Conclusion

Across a range of research projects representing diverse regions and populations in the US, we observed multiple strategies for leveraging self-sampling and mail for CRC screening, from organized FIT outreach to opportunistic offers for FIT or FIT-DNA with mailed return. Furthermore, 7 of 8 projects successfully implemented mailed FIT outreach, including the use of nearly every best practice strategy for mailed FIT implementation. Our observations suggest that great potential remains for more broadly leveraging mail and self-sampling for cancer screening, including with mailed FIT outreach. Additionally, this work serves as a foundation for future ACCSIS research that can compare how outcomes of screening promotion (ie, screening and follow-up colonoscopy participation) differ by implementation strategies used. Understanding relative performance of different implementation strategies could help optimize self-sampling–based cancer screening for CRC and other screening and diagnostic tests.

## Appendix

The ACCSIS consortium developed a framework, which is divided into 3 phases: pre-implementation, implementation, and postimplementation. The pre-implementation phase centers on choosing interventions, describing the context in which the interventions are implemented, and preparing for implementation. The implementation phase describes the interventions and outcomes at the patient, provider, clinic, and community levels and outlines the short-term and long-term screening outcomes. Intervention impact analysis, equity assessments, and economic evaluations are in the post-implementation phase, as are dissemination of findings, intervention maintenance, and intervention scalability. For this article, we identified the following framework elements from the phases of each ACCSIS Research Project: socioecological context, program characteristics, implementation strategies, and evaluation outcomes.
